# Statistical tests for non-independent partitions of large autocorrelated datasets

**DOI:** 10.1016/j.mex.2022.101660

**Published:** 2022-03-12

**Authors:** Anthony R. Ives, Likai Zhu, Fangfang Wang, Jun Zhu, Clay J. Morrow, Volker C. Radeloff

**Affiliations:** Integrative Biology, University of Wisconsin-Madison, Madison, WI 53706, USA

**Keywords:** Statistics for big data, Remote sensing, Hypothesis tests on large datasets, Likelihood ratio test, F-test, t-test

## Abstract

Large sets of autocorrelated data are common in fields such as remote sensing and genomics. For example, remote sensing can produce maps of information for millions of pixels, and the information from nearby pixels will likely be spatially autocorrelated. Although there are well-established statistical methods for testing hypotheses using autocorrelated data, these methods become computationally impractical for large datasets.

• The method developed here makes it feasible to perform *F*-tests, likelihood ratio tests, and *t*-tests for large autocorrelated datasets. The method involves subsetting the dataset into partitions, analyzing each partition separately, and then combining the separate tests to give an overall test.

• The separate statistical tests on partitions are non-independent, because the points in different partitions are not independent. Therefore, combining separate analyses of partitions requires accounting for the non-independence of the test statistics among partitions.

• The methods can be applied to a wide range of data, including not only purely spatial data but also spatiotemporal data. For spatiotemporal data, it is possible to estimate coefficients from time-series models at different spatial locations and then analyze the spatial distribution of the estimates. The spatial analysis can be simplified by estimating spatial autocorrelation directly from the spatial autocorrelation among time series.

## Graphical abstract

**Figure:**
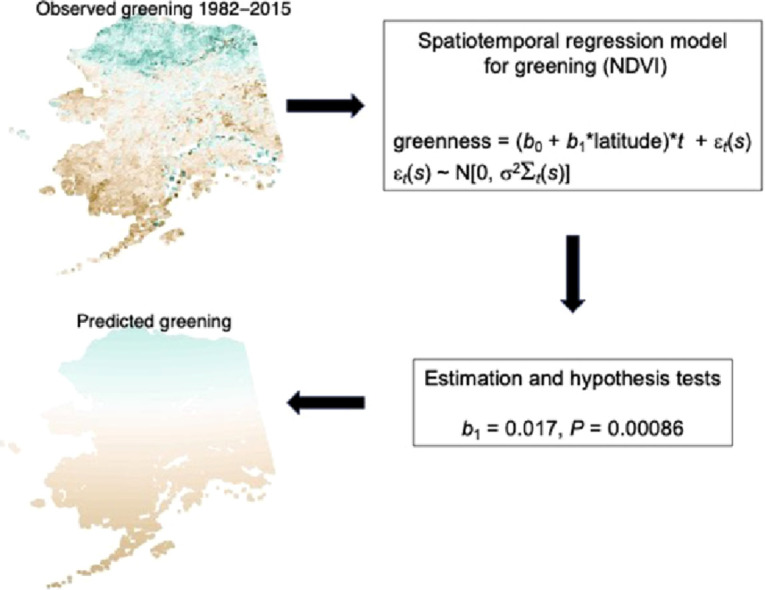

Specifications Table

**Table:** 

Subject Area;	Environmental Science
More specific subject area;	*Statistics*
Method name;	*Method for performing statistical tests using non-independent data partitions*
Name and reference of original method;	*These are standard statistical methods documented in most statistical textbooks, for example, Neter et al.*[12]*and Judge et al.*[10]
Resource availability;	*The methods are implemented in the package remotePARTS in the R programming language. This is available at*https://github.com/morrowcj/remotePARTS

## Method details

### Overview

The method developed here uses a conceptually simple approach to perform statistical estimation and hypothesis tests on large autocorrelated datasets [1,4,8,11,13,14]. The method was developed specifically for remote-sensing datasets, so it will be described in this context, although it could be applied to other types of large datasets such genome samples from different subjects in which base-pair similarity is likely to be greater for base pairs located nearby on a chromosome due to limited recombination. The approach divides the dataset into non-overlapping partitions, and statistical hypothesis tests are conducted on each partition. The results of these tests are not independent, because the data points from different partitions are not independent. Nonetheless, it is possible to calculate the correlation between the statistical test scores from different partitions and combine the scores for an overall test. The selection of sampling schemes for partitions is arbitrary, but here we will focus on random sampling to produce partitions. This approach has the advantage of guaranteeing that each partition gives a representation of the entire dataset. The method is central to the analysis of spatiotemporal data given in Ives et al. [9].

We apply this approach to three common tests used for regression and ANOVA on Gaussian data [12]: the *F*-test, the likelihood ratio test (LRT), and the *t*-test. The *F*-test and LRT involve hypotheses comparing a full statistical model with a reduced model, thereby allowing tests of hypotheses on multiple coefficients in a model. The *t*-test is used for inference about individual coefficients. These tests can be performed for datasets with autocorrelated errors using generalized least squares (GLS) regression [10]. In GLS, a correlation matrix is specified to describe the autocorrelation of errors. We apply the approach to both purely spatial and spatiotemporal data.

### Mathematical derivations

The specific problem addressed here involves the regression of a response (dependent) variable *y_l_* on *p* predictor (independent) variables for *l* = 1, 2, ..., *N* locations in a spatial map. The *p* predictor variables for location *l* are contained in a 1 x *p* vector *X_l_*; at the minimum, *X_l_* contains the value 1 to correspond to an intercept. The regression model for location *l* is

(1) $$y_{l}=X_{l}B\;+\;\gamma_{l}$$

where γ*_l_* is the remaining random error, and **B** is a *p* x 1 vector containing regression coefficients for the *p* variables in *X_l_*. We assume that the parameters **B** are the same for all locations *l*. We further assume that the correlation between γ*_l_* and γ*_k_* for locations *l* and *k* depends on the distance between them. For example, if the correlation diminishes exponentially with distance, then cor[γ*_l_*, γ*_k_*] = exp(–*d_lk_*/*r*) where *d_lk_* is the distance between locations *l* and *k*, and the parameter *r* gives the "range" of the correlation, with larger values of *r* giving correlations over greater spatial distances. Thus, the covariance matrix for the errors, **Γ** = (γ_1_, ..., γ*_N_*)′ (where the apostrophe denotes transpose), is the *N* x *N* covariance matrix **V** = σ^2^**C**, where **C** is the correlation matrix containing the values of cor[γ*_l_*, γ*_k_*] for all pairs of locations *l* and *k*, and σ^2^ is a common variance.

### GLS analysis

A single dataset, or a subset of a larger dataset, in which errors are autocorrelated can be analyzed by GLS when **V** is known [10]. Thus, let **Y** denote an *N* x 1 vector of response variables, and **X** denote an *N* x *p* matrix of predictor variables including a column of ones for the intercept. The *p* x 1 vector $\hat{B}$ containing the estimates of the regression coefficients **B** is

(2) $$\hat{B}={\left(X\prime V^{-1}X\right)}^{-1}\left(X\prime V^{-1}Y\right)$$

The sum-of-squared error is then

(3) $$SSE={\left(Y-X\hat{B}\right)}^{\prime }V^{-1}\left(Y-X\hat{B}\right)$$

An *F*-test is based on the sum-of-squared error of the full model (*SSE*) and a reduced model (*SSE*_0_) defined by the hypothesis to be tested; for example, the null hypothesis that some of the predictor variables have no effect on the response variable is tested using the reduced model with these predictor variables removed. Under the null hypothesis, and letting *SSR*_1–0_ = *SSR*_1_ – *SSR*_0_ = *SSE*_0_ – *SSE* denote the difference in sum-of-squared regression between full and reduced models, we have

(4) $$\left(\mathrm{SS}R_{1-0}/df_{1}\right)/\left(\mathrm{SSE}/df_{2}\right)∼\;F$$

Thus, the ratio of *SSR*_1–0_/*df*_1_ to *SSE*/*df*_2_ follows an *F* distribution where *df*_1_ is the degrees of freedom equaling the difference in the number of predictor variables between full and reduced models, and *df*_2_ = *N* – *p* is the degrees of freedom for the sum-of-squared error in the full model. Strategic selection of the reduced model makes it possible to test a wide range of hypotheses, not only about whether one or more of the coefficients in **B** are different from zero, but also whether some or all of the coefficients are equal by generating orthogonal contrasts [12].

For GLS, a LRT is closely related to an *F*-test. Specifically, the log-likelihood ratio between the full and reduced models is *SSR*_1–0_ for the case when the full and reduced models have the same covariance matrix **V**. The LRT uses the asymptotic approximation

(5) $$2\;SSR_{1-0}\;∼\;\chi_{df1}$$

Thus, twice the difference between the *SSR*s from the full and reduced models is χ^2^ distributed with *df*_1_ degrees of freedom. The distribution for the *F*-test (Eq. 4) will converge to the distribution for the LRT when *df*_2_ approaches infinity. For large datasets, *df*_2_ will be large, and the *F*-test and LRT will give very similar results.

Like the LRT, the *t*-test is closely related to the *F*-test. Letting *MSE* = *SSE*/*df*_2_ be the mean squared error, the estimated covariance matrix for the estimates $\hat{B}$ is

(6) $$\hat{\text{var}}\left[\hat{B}\right]\;=\;MSE\;{\left(X\prime V^{-1}X\right)}^{-1}$$

The standard error of the estimate of a coefficient *b_h_* contained in $\hat{B}$, se[$\hat{b}$*_h_*], is the square-root of the *h*^th^ diagonal element of $\hat{\text{var}}$ [$\hat{B}$], and a test of the null hypothesis that the coefficient is zero is performed with *t*-distribution:

(7) $${\hat{b}}_{h}/\text{se}\left[{\hat{b}}_{h}\right]\;∼\;t_{df2}$$

To simplify the following developments, it is useful to recast the GLS model above by transforming variables. Specifically, let **D** be the matrix such that **DVD′** = **I**. For example, **D** could be the inverse of the Cholesky decomposition of **V**. With this construction of **D**, the covariance matrix for the transformed errors **A** = **DΓ** is E[**AA′**] = E[(**DΓ**)(**DΓ**)′] = **DVD′** = **I**. If **U** = **DX** and **Z** = **DY**, then

(8) $$\hat{B}\;=\;{\left(U\text{'}U\right)}^{-1}\left(U\text{'}Z\right)$$

Further, let **H** denote the hat matrix for **U** defined by

(9) $$H\;=\;U{\left(U\prime U\right)}^{-1}U\prime$$

Then

(10) $$SSR_{1-0}=\;Z\prime \left(H-H_{0}\right)Z$$

(11) SSE=Z′(I−H)Z

where **H_0_** is the hat matrix for the reduced model derived from **U_0_** = **DX_0_**, and **X_0_** is the matrix of predictor variables in the reduced model.

### *Correlations among partitions for* SSR*_1–0_,* SSE*, and*$\hat{B}$

The primary computational burden of GLS is inverting **V** (or its Cholesky decomposition). A map with 10^6^ pixels would require inverting a 10^6^ x 10^6^ **V** matrix, and the computation time for this inversion scales roughly with *N*^3^. If a map is partitioned into *n_p_* subsets of size *m* and each subset is analyzed separately, then the computational burden will scale linearly with *N* for a fixed subset size *m*. Using the computational advantage of analyzing partitions, the method below makes it feasible to combine the results from the *n_p_* separate analyses.

For the *F*-test, the method requires calculating the correlation between sums-of-squares computed for the *n_p_* different subsets. Specifically, it is necessary to calculate the correlation between *SSR_i_* and *SSR_j_*, and between *SSE_i_* and *SSE_j_*; for notational convenience, *SSR_i_* denotes *SSR*_1–0_ (the difference between the SSRs for the full and reduced models) for partition *i*, and *SSE_i_* denotes the *SSE* of the full model for partition *i.* Further, let **Z***_i_*, **U***_i_*, and **A***_i_* denote the transformed response and predictor variables, and the transformed errors, for partition *i*. It is possible to show that, for any *m* x *m* matrices **S***_i_* and **S***_j_*,

(12) $$\text{cov}\left[\left({A\prime }_{i}S_{i}A_{i}\right)\left({A\prime }_{j}S_{j}A_{j}\right)\right]\;=\;vec\left(S_{i}\right)\prime \text{cov}\left[\left(A_{i}{A\prime }_{i}\right)⊗\left(A_{j}{A\prime }_{j}\right)\right]vec\left(S_{j}\right)$$

where *vec* is the vec operator that stacks columns of a matrix on top of each other to form a vector, and ⊗ is the Kronecker product. The matrix cov[(**A***_i_***A***_i_***′**)⊗(**A***_j_***A***_j_***′**)] can be expressed in terms of the matrix **V***_ij_* = σ^2^**C***_ij_* containing covariances between errors γ*_i_*_,_*_l_* and γ*_j_*_,_*_k_* from partitions *i* and *j*, respectively. Specifically, cov[(**A***_i_***A***_i_***′**)⊗(**A***_j_***A***_j_***′**)] = **R***_ij_*⊗**R***_ij_* + **Ρ**, where **R***_ij_* = **D***_i_***C***_ij_***D***_j_***′** and **Ρ** is the matrix constructed by horizontally joining the matrices **R***_ij_*⊗**R***_ij_*_[φ]_ where [φ] denotes the φ^th^ column of **R***_ij_*. Letting **H***_i_* and **H_0_***_i_* denote the hat matrices **H***_i_* = **U***_i_*(**U***_i_***′U***_i_*)^–1^**U***_i_***′** and **H_0_***_i_* = **U_0_***_i_*(**U_0_***_i_***′U_0_***_i_*)^–1^**U_0_***_i_***′**, we have

(13) $$\text{cor}\left[SSR_{i},SSR_{j}\right]\;=\;vec{\left(H_{i}-H_{0i}\right)}^{\prime }\left(R_{ij}⊗R_{ij}\right)vec\left(H_{j}-H_{0j}\right)/df_{1}$$

where the appearance of *df*_1_ arises by noting that var[*SSR_i_*] = *vec*(**H***_i_* – **H_0_***_i_*)**′***vec*(**H***_i_* – **H_0_***_i_*) = 2*df*_1__._ Eq. (13) was simplified using the empirically confirmed identity that *vec*(**H***_i_* – **H_0_***_i_*)**′**(**R***_ij_* ⊗ **R***_ij_*) *vec*(**H***_j_* – **H_0_***_j_*) = *vec*(**H***_i_* – **H_0_***_i_*)**′Ρ***vec*(**H***_j_* – **H_0_***_j_*) for the matrices **H***_j_* – **H_0_***_j_* under the null hypothesis. Using a similar derivation,

(14) $$\text{cor}\left[SSE_{i},SSE_{j}\right]=vec{\left(I-H_{i}\right)}^{\prime }\left(R_{ij}⊗R_{ij}\right)vec\left(I-H_{j}\right)/df_{2}$$

The LRT depends on the *SSR*_1–0_, and therefore the method requires calculating the correlations between values of *SSR_i_* from the *n_p_* partitions, as is already given for the *F*-test. For *t*-tests on the coefficient values, it is necessary to calculate the correlations between the estimators of the coefficients given by Eq. (8), which are given as

(15) $$\text{cor}\left[{\hat{B}}_{i},{\hat{B}}_{j}\right]={\left({U\prime }_{i}U_{i}\right)}^{-1}\;\left({U\prime }_{i}R_{ij}U_{j}\right){\left({U\prime }_{j}U_{j}\right)}^{-1}$$

where $\hat{B}$*_i_* is the estimator of the coefficients from partition *i*.

### Combining tests from the partitions

From the values of *SSR_i_* and *SSE_i_* calculated for each partition, *i* = 1, 2, ..., *n_p_*, and the correlations between *SSR_i_* and *SSR_j_*, and between *SSE_i_* and *SSE_j_*, it is possible to compute an overall *F*-test for the data. The procedure for the LRT is similar to that for the *F*-test, and below they are presented together. The procedure for the *t*-test is somewhat different and is described after the *F*-test and LRT.

For a given partition *i*, 2*SSR_i_* follows a χ^2^ distribution with *df*_1_ degrees of freedom. A χ^2^ distribution with *df*_1_ degrees of freedom is the sum of *df*_1_ squared Gaussian variables with mean 0 and variance 1. Thus, *SSR_i_* can be expressed as *SSR_i_* = *G*^2^*_i,_*_1_ + *G*^2^*_i,_*_2_  + ... + *G*^2^*_i_*_,_*_df_*_1_. The overall test statistic depends on the sum of *SSR_i_* from all partitions *i* = 1, 2, ..., *n_p_*. Let **G** denote the (*n_p_ df*_1_) x 1 vector of values of *G_i_*_,μ_ (μ = 1, ..., *df*_1_) from all partitions. By construction Eqs. 8-(11), the distributions *G_i,_*_μ_ and *G_i,_*_ν_ (μ ≠ ν) within the same partition *i* are independent. To satisfy the identity in Eq. (13) for *SSR*, the correlations between *G_i,_*_μ_ and *G_j,_*_ν_ from different partitions *i* and *j* are

(16) $$\text{cor}\left[G_{i,\mu },G_{j,\nu }\right]={\left(\rho_{i,j}/df_{1}\right)}^{1/2}$$

for ρ*_i,j_* = *vec*(**S***_i_*)**′**(**R***_ij_*⊗**R***_ij_*)*vec*(**S***_j_*)/*df*_1_ and **S***_i_* = (**H***_i_* – **H_0_***_i_*). Letting **P** be the correlation matrix containing values of cor[*G_i,_*_μ_, *G_j,_*_ν_], the distribution of the sum of *SSR_i_* from all partitions is

(17) $$SSR=\sum_{i}SSR_{i}\;∼\;G\prime \mathrm{PG}$$

where **G**′**PG** follows a quadratic Gaussian distribution. The probability density function of this quadratic Gaussian can be computed directly [5,7] to give the probability of **G′PG** being greater than an observed value of *SSR*, which produces the *P*-value of the LRT.

The *F*-test depends on both *SSR* and *SSE*, where *SSE* is defined like *SSR* as *SSE* = Σ*_i_SSE_i_* and each *SSE_i_* has *df*_2,_*_i_* degrees of freedom. Because partitions may differ in size, *df*_2,_*_i_* may differ among partitions. Because the values of *df*_2,_*_i_* will be large (certainly >100), the values of *SSE_i_*/*df*_2,_*_i_* will be approximately Gaussian distributed with mean 1 and variance 2/*df*_2,_*_i_*. The correlation between the Gaussian distributions of *SSE_i_*/*df*_2,_*_i_* and *SSE_i_*/*df*_2,_*_i_* can be derived from Eq. (14) with **S***_i_* = (**I** – **H***_i_*). Thus, the *F*-score is approximated as the quadratic Gaussian distribution for *SSR* divided by the Gaussian distribution for *SSE*. There is no closed-form expression for this distribution, and therefore it is obtained via simulating a large number (e.g., 10^5^) values to generate the approximate (parametric bootstrapped) test distribution.

For the *t*-test, the estimator of the coefficient *b_h,i_*, $\hat{b}$*_h,i_*, from partition *i* follows a Gaussian distribution, and the test statistic for the mean value of $\hat{b}$*_h,i_* from all partitions is (Σ*_i_*$\hat{b}$*_h,i_*/*n_p_*)/se[Σ*_i_*$\hat{b}$*_h,i_*/*n_p_*] Eq. (15). gives the correlation between $\hat{b}$*_h,i_* and $\hat{b}$*_h,j_* from partitions *i* and *j*, cor[$\hat{b}$*_h,i_*, $\hat{b}$*_h,j_*]. Thus, se[Σ*_i_*$\hat{b}$*_h,i_*/*n_p_*]^2^ = (1/*n_p_*)^2^Σ*_ij_*cor[$\hat{b}$*_h,i_*, $\hat{b}$*_h,j_*]se[$\hat{b}$*_h,i_*]se[$\hat{b}$*_h,j_*] is the sum of *n_p_* random variables each having a χ^2^ distribution with *df*_2,_*_i_* degrees of freedom. From this expression, (Σ*_i_*$\hat{b}$*_h,i_*/*n_p_*)/se[Σ*_i_*$\hat{b}$*_h,i_*/*n_p_*] is approximately distributed as a *t*-distribution with Σ*_i_df*_2,_*_i_* degrees of freedom; for large degrees of freedom *df*_2,_*_i_*, this will approach a Gaussian distribution with mean zero and variance one.

### Spatiotemporal analyses

The spatiotemporal analyses follow the approach presented in Ives et al. [9] for analyzing time trends in remote-sensing data. The approach involves first fitting a time-series model to the time series in each pixel on a map and obtaining the estimate of the time trend. As described below, the correlations between the residuals obtained from the fitted time-series model approximate the spatial autocorrelation of the estimated coefficients of the time trends. Therefore, the spatial autocorrelation matrix required for the GLS spatial analysis can be estimated before the GLS analysis is performed. Although this approach can be used for different time-series models, here we focus on two: least-squares (LS) regression, and regression with AR(1) errors estimated using REML. The former is useful, because it allows analytical solutions, whereas the second has better statistical properties and is therefore preferentially used for the analyses in Ives et al. [9].

To explain the approach, we use a specific spatiotemporal model, and we also use this model in the validation. The model for time series within pixel *l* is

$$z_{l}\left(t\right)=a_{l}+\;c_{l}t\;+\;\varepsilon_{l}\left(t\right)$$

(18) $$\varepsilon_{l}\left(t\right)=\beta_{l}\varepsilon_{l}\left(t-1\right)+\delta_{l}\left(t\right)$$

Here, *z_l_*(*t*) is the value of interest in pixel *l* at time *t* (*t* = 1, 2, ..., *T*), *a_l_* is the intercept, and *c_l_* is the time trend. Random errors ε*_l_*(*t*) follow a stationary first-order Gaussian autoregressive process with mean zero generated from the Gaussian random variable δ*_l_*(*t*) that has mean zero and variance σ^2^, with values independent through time so that E[δ*_l_*(*t*) δ*_l_*(*s*)] = 0 for *s* ≠ *t*. Thus, the vector (ε*_l_*(1) , ..., ε*_l_*(*T*))′,has distribution N(0, σ^2^/(1 – β*_l_*^2^) **Σ***_l_*), where the elements of the correlation matrix **Σ***_l_* are cor[ε*_l_*(*t*), ε*_l_*(*s*)] = β*_l_*^|^*^t^*^–^*^s^*^|^ for all *t* and *s*. To include spatial autocorrelation, we assume that the Gaussian random variables δ*_l_*(*t*) and δ*_k_*(*t*) at *t* from pixels *l* and *k* are correlated, with parameter corδ = cor[δ*_l_*(*t*), δ*_k_*(*t*)], but values of δ*_l_*(*t*) and δ*_k_*(*s*) are independent when *s* ≠ *t*.

The procedure for spatiotemporal data collapses temporal information from the time series into two quantities: the pixel-specific estimates of the time trend *c_l_* and the correlations between the temporal errors ε*_l_*(*t*) and ε*_k_*(*t*) from pixels *l* and *k*. Note that the estimates of *c_l_*, $\hat{c}$*_l_*, are now the dependent variable in the spatial model, rather than the data *z_l_*(*t*). For LS regression, the exact relationship between the correlation cor[$\hat{c}$*_l_*, $\hat{c}$*_k_*] and cor[ε*_l_*(*t*), ε*_k_*(*t*)] can be derived analytically under the assumption that the temporal autocorrelation coefficients β*_l_* and β*_k_* are known. The *T* x *T* covariance matrix **W***_lk_* whose *t,s*-element (*t* = 1, ..., *T; s* = 1, ..., *T*) is the covariance between ε*_l_*(*t*) and ε*_k_*(*s*) is given by

(19) $$W_{lk}=\text{co}r_{\delta }\;{\left(I-\beta_{i}\Psi \right)}^{-1}\;\Delta_{lk}{\left({\left(I-\beta_{k}\Psi \right)}^{-1}\right)}^{\prime }$$

where **I** is the *T* x *T* identity matrix, and **Ψ** is the backward shift operator [3]. Under the assumption that the time series are sampled from their stationary distributions, **Δ***_lk_* is a diagonal matrix whose first diagonal element is the value of cov[ε*_l_*(*t*), ε*_k_*(*t*)] at the stationary distribution, and other diagonal elements are one. The stationary distribution of **Ε**(*t*) = (ε*_l_*(*t*), ε*_k_*(*t*)), follows a bivariate Gaussian distribution with mean (0, 0), and covariance matrix **Ω_Ε_** satisfying vec(**Ω_Ε_**) = σ^2^(**I** – **Φ**⊗**Φ**)^–1^*vec*(**Ω**) where **Φ** is the 2 x 2 diagonal matrix containing β*_l_* and β*_k_*, and **Ω** is the 2 x 2 matrix with 1 on the diagonal and corδ on the off-diagonal.

For LS regression, cor[$\hat{c}$*_l_*, $\hat{c}$*_k_*] can be computed as

(20) $$\text{cor}\left[{\hat{c}}_{l},{\hat{c}}_{k}\right]=\left(KW_{\mathrm{lk}}K^{\text{'}}\right)/\left(\left(KW_{\mathrm{ll}}K^{\text{'}}\right)\left(KW_{\mathrm{kk}}K^{\text{'}}\right)\right)$$

where **K** is the second row of the 2 x *T* matrix (**J′J**)^–1^**J**, where **J** is the *T* x 2 matrix containing 1 in the first column and time *t* = 1, ..., *T* in the second column; in other words, **J** is the matrix of independent variables that would be used to fit Eq. (18) using LS regression.

We used Eqs. (19) and (20) to explore the relationship between cor[$\hat{c}$*_l_*, $\hat{c}$*_k_*] and cor[ε*_l_*(*t*), ε*_k_*(*t*)], thereby investigating the validity of using cor[ε*_l_*(*t*), ε*_k_*(*t*)] as an approximation of cor[$\hat{c}$*_l_*, $\hat{c}$*_k_*] in a spatial GLS analysis of $\hat{c}$*_l_*. Eqs. (19) and (20) apply only for LS regression analyses of the time series (Eq. 18), and therefore we performed simulations for regression with AR(1) errors estimated using REML. When β*_l_* = β*_k_*, cor[$\hat{c}$*_l_*, $\hat{c}$*_k_*] = cor[ε*_l_*(*t*), ε*_k_*(*t*)] = corδ for LS regression (Table 1). For AR(1) regression, cor[$\hat{c}$*_l_*, $\hat{c}$*_k_*] and cor[ε*_l_*(*t*), ε*_k_*(*t*)] are slightly lower than corδ, although they are equal (within the uncertainty of the simulation) (Table 2). Therefore, when β*_l_* = β*_k_*, cor[ε*_l_*(*t*), ε*_k_*(*t*)] is an excellent approximation for cor[$\hat{c}$*_l_*, $\hat{c}$*_k_*]. Keeping β*_l_* = 0.8, as β*_k_* decreases from the high value of β*_k_* = 0.8, cor[ε*_l_*(*t*), ε*_k_*(*t*)] decreases below corδ; cor[$\hat{c}$*_l_*, $\hat{c}$*_k_*] also decreases below corδ, but the decrease is not as great as cor[ε*_l_*(*t*), ε*_k_*(*t*)] especially for longer time series (larger *T*). Therefore, cor[$\hat{c}$*_l_*, $\hat{c}$*_k_*] is generally greater than cor[ε*_l_*(*t*), ε*_k_*(*t*)], with the ratio cor[$\hat{c}$*_l_*, $\hat{c}$*_k_*]/cor[ε*_l_*(*t*), ε*_k_*(*t*)] greater when the difference between β*_l_* and β*_k_* is large, and when the time-series are longer. For the example dataset from Alaska analyzed in Ives et al. [9], the time series have length *T* = 34, and estimates of β*_l_* had a mean of 0.40 and a standard deviation of 0.23. The simulations in Table 2 for AR(1) regression suggest that using cor[ε*_l_*(*t*), ε*_k_*(*t*)] to approximate cor[$\hat{c}$*_l_*, $\hat{c}$*_k_*] will lead to a small overestimate of cor[$\hat{c}$*_l_*, $\hat{c}$*_k_*], although this will not change the conclusions; this is discussed in detail in Ives et al. [9].

**Table 1:** Relationship between the correlation for estimates of the time trend parameter, cor[$\hat{c}$*_l_*, $\hat{c}$*_k_*], and the correlation for residuals from least-squares (LS) regression, cor[ε*_l_*(*t*), ε*_k_*(*t*)]. To explore extreme differences in the strength of temporal autocorrelation between time series, β*_l_* = 0.8 for one time series and β*_k_* = –0.4, 0, 0.4, and 0.8 for the other time series, with time-series length *T* = 10, 30, or 100. The values of cor[ε*_l_*(*t*), ε*_k_*(*t*)] and cor[$\hat{c}$*_l_*, $\hat{c}$*_k_*] were calculated analytically using Eqs. (19) and (20), respectively. Throughout, the parameter governing the correlation between δ*_l_*(*t*) and δ*_l_*(*s*), corδ, was 0.5.

β*_l_*	β*_k_*	*T*	cor_δ_	cor[ε*_l_*(*t*), ε*_k_*(*t*)]	$\mathrm{cor}[{\hat{c}}_{l},{\hat{c}}_{k}]$	$\mathrm{cor}[{\hat{c}}_{l},{\hat{c}}_{k}]$/cor[ε*_l_*(*t*), ε*_k_*(*t*)]
0.8	-0.4	10	0.5	0.21	0.25	1.21
0.8	-0.4	30	0.5	0.21	0.38	1.84
0.8	-0.4	100	0.5	0.21	0.46	2.22
0.8	0.0	10	0.5	0.30	0.32	1.06
0.8	0.0	30	0.5	0.30	0.41	1.37
0.8	0.0	100	0.5	0.30	0.47	1.58
0.8	0.4	10	0.5	0.40	0.41	1.00
0.8	0.4	30	0.5	0.40	0.45	1.11
0.8	0.4	100	0.5	0.40	0.48	1.20
0.8	0.8	10	0.5	0.50	0.50	1.00
0.8	0.8	30	0.5	0.50	0.50	1.00
0.8	0.8	100	0.5	0.50	0.50	1.00

**Table 2:** Relationship between the correlation for estimates of the time trend parameter, cor[$\hat{c}$*_l_*, $\hat{c}$*_k_*], and the correlation for residuals, cor[ε*_l_*(*t*), ε*_k_*(*t*)], from regression with AR(1) errors fit with REML. To explore extreme differences in the strength of temporal autocorrelation between time series, β*_l_* = 0.8 for one time series and β*_k_* = –0.4, 0, 0.4, and 0.8 for the other time series, with time-series length *T* = 10, 30, or 100. The values of cor[ε*_l_*(*t*), ε*_k_*(*t*)] and cor[$\hat{c}$*_l_*, $\hat{c}$*_k_*] were calculated by simulating Eq. (18) for 50,000 pairs of time series. Throughout, the parameter governing the correlation between δ*_l_*(*t*) and δ*_l_*(*s*), cor_δ_, was 0.5.

β*_l_*	β*_k_*	*T*	cor_δ_	cor[ε*_l_*(*t*), ε*_k_*(*t*)]	cor[$\hat{c}$*_l_*, $\hat{c}$*_k_*]	cor[$\hat{c}$*_l_*, $\hat{c}$*_k_*]/cor[ε*_l_*(*t*), ε*_k_*(*t*)]
0.8	-0.4	10	0.5	0.23	0.24	1.04
0.8	-0.4	30	0.5	0.22	0.33	1.54
0.8	-0.4	100	0.5	0.21	0.43	2.06
0.8	0.0	10	0.5	0.31	0.30	0.95
0.8	0.0	30	0.5	0.31	0.37	1.19
0.8	0.0	100	0.5	0.30	0.44	1.47
0.8	0.4	10	0.5	0.41	0.40	0.97
0.8	0.4	30	0.5	0.40	0.41	1.01
0.8	0.4	100	0.5	0.40	0.47	1.16
0.8	0.8	10	0.5	0.46	0.49	1.06
0.8	0.8	30	0.5	0.48	0.47	0.98
0.8	0.8	100	0.5	0.49	0.48	0.99

To build the correlation matrix **C** for the GLS spatial analysis, we assume that cor[$\hat{c}$*_l_*, $\hat{c}$*_k_*] decays according to some function *v*(*d_lk_*) of the distance *d_lk_* between pixels *l* and *k*. For example, Ives et al. [9]use an exponential-power function *v*(*d_lk_*) = exp(-(*d_lk_*/*r*)*^g^*) where the range parameter *r* and shape parameter *g* are estimated from the calculated values of cor[ε*_l_*(*t*), ε*_k_*(*t*)] for a subset of pairs of locations using nonlinear regression of cor[ε*_l_*(*t*), ε*_k_*(*t*)] on *d_lk_*. For large datasets, the estimates of parameters in *v*(*d_lk_*) have small standard errors, and therefore uncertainty in *v*(*d_lk_*) is assumed to be negligible.

### Implementation

The partition approach for performing statistical tests can be implemented directly using the derivations above. When using random partitions, for large datasets it is not necessary to calculate the pairwise correlations between *SSR_i_* (or *SSE_i_*) from all partitions Eqs. 13,(14), or the pairwise correlations between the estimates of the coefficients from all partitions (Eq. 15). These correlations vary little between different pairs of partitions, and the number of partitions can be set after examining the variation in correlations for specific datasets. This approach saves considerable computational time for large datasets with many partitions.

Although the methods are presented assuming that the correlation matrices **C*_i_*** are known, in many applications **C*_i_*** will contain one or more parameters to be estimated. For example, spatial models will often contain a "nugget" to capture local (non-spatial autocorrelation) variation [4,9]. In the spatiotemporal analysis we outline above, other parameters giving the spatial extent of the autocorrelation (e.g., *r* in *v*(*d_lk_*)) can be estimated from the residuals of the time-series analyses. However, for the purely spatial model these would be estimated during the GLS spatial analysis. Any parameters of **C*_i_*** can be estimated for each partition separately and the formulae applied with the estimated matrices of **C*_i_***. The resulting omnibus test statistics are then conditional on the parameter estimates of **C*_i_***.

The methods are implemented as a part of the package remotePARTS in the R programming language. It is available at https://github.com/morrowcj/remotePARTS.

## Methods validation

### Spatial model

To assess type I error rates and power, we performed a simulation study using the regression model

(21) $$y_{l}=b_{0}+\;bx_{l}\;+\;\gamma_{l}$$

in which the *N* spatial errors γ*_l_* follow a multivariate Gaussian distribution with correlation matrix **C**, N(0, σ^2^_γ_**C**). The simulation was performed on a 60 x 60 pixel map (*N* = 3,600), which was small enough to perform a GLS analysis Eqs. 1-(7) without partitioning the datasets. Spatial autocorrelation was introduced by assuming that the elements of **C** equal exp(–*d_lk_*/*r*). Values of *r* were standardized to the scale of the map, and values of *r* = 0.03 and 0.1 correspond to 3% and 10% of the maximum distance on the map (corner to corner). Values of *x_l_* were given by the “latitude” that was defined as the row number in the 60 x 60 map divided by 60. For each value of *b* = 0, 2, 4, ..., 20, five hundred simulations were performed, and the null hypothesis H_0_:*b* = 0 was tested for each at the significance levels of α = 0.05 and 0.01. We performed the partition analyses with eight partitions, and computed the pairwise correlations between *SSR_i_* (or *SSE_i_*) Eqs. 13,(14) as the average from a random subset of six partitions (a subset of 15 of the 28 total number of pairwise correlations).

**Fig. 1:**
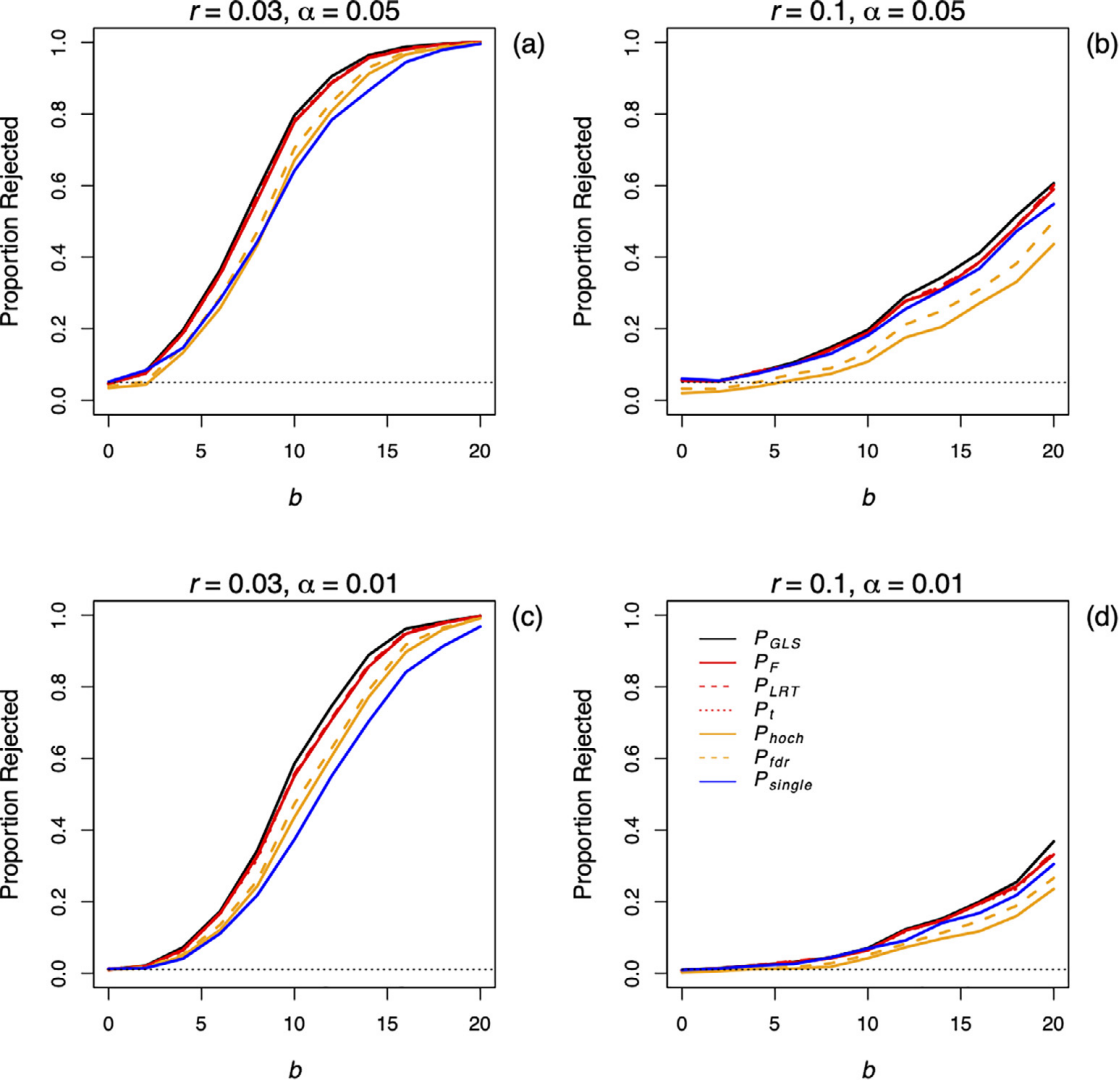
Type I errors and power for the methods combining correlated tests among partitions for spatial data. Five-hundred simulations were performed on a 60 x 60 map using equation (19) for each value of *b* = 0, 2, 4, ..., 20, with either moderate (*r* = 0.03; a,c) or strong (*r* = 0.1; b,d) spatial autocorrelation. The hypothesis H_0_:*b* = 0 was tested at significance levels of α = 0.05 (a,b) and 0.01 (c,d) using: an GLS *F*-test (*P_GLS_*); the partition method with eight partitions giving an *F*-test (*P_F_*), a LRT (*P_LRT_*), and a *t*-test (*P_t_*); selecting the lowest *P*-value and applying a Hochberg (*P_hoch_*) or the False Discovery Rate adjustment (*P_fdr_*); and randomly selecting one partition (*P_single_*).

Fig. 1 gives the proportion of the simulations for which H_0_:*b* = 0 was rejected, with the significance level α given by the black dotted line. The tests were based on different methods for producing *P*-values. First, GLS (Eq. 4) was used to perform an *F*-test (*P_GLS_*), which gives the "gold standard" since the GLS is the best linear unbiased estimator (BLUE) [10]. Second, the partition method developed here was applied using eight partitions to give an *F*-test (*P_F_*), a LRT (*P_LRT_*), and a *t*-test (*P_t_*). Third, the lowest *P*-value from the eight partitions from *F*-tests was selected and adjusted for eight multiple comparisons using either the Hochberg adjustment (*P_hoch_*) [6] or the False Discovery Rate adjustment (*P_fdr_*) [2]. Finally, a single partition was selected at random and its *P*-value was used (*P_single_*).

As expected, all three values using the method developed here (*P_F_, P_LRT_*, and *P_t_*) gave very similar results. Type I error rates were appropriate, with close to 5% and 1% of simulations rejected at significance levels of α = 0.05 and 0.01, respectively. There was slight loss of power in comparison to the GLS (*P_GLS_*). Nonetheless, this loss of power was less than that of either Hochberg or FDR adjustments for multiple comparisons (*P_hoch_* and *P_fdr_*). In fact, when autocorrelation was high (*r* = 0.1), the Hochberg or FDR adjustments had lower power than the randomly chosen partition (*P_single_*).

**Fig. 2:**
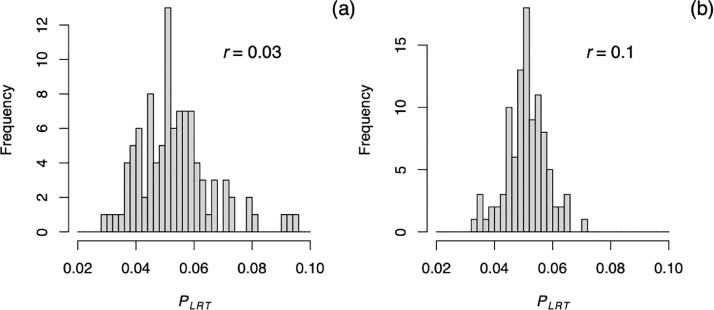
*P*-values from a LRT of H_0_:*b* = 0 applied to the same simulated 60 x 60 pixel dataset but using different random partitions. (a) A simulated dataset with *b* = 20 and the range parameter *r* = 0.03, and (b) a simulated dataset with *b* = 5 and the range parameter *r* = 0.1. The example datasets were selected to give *P*-values near 0.05. For each panel, the same dataset was fit 100 times with different partitions. Eight random partitions each containing 450 pixels were selected for each analysis.

Because partitions were created randomly, there is variation in the test statistics depending on the partitions created. To illustrate this, Fig. 2 gives the distributions of *P*-values for a partition LRT of H_0_:*b* = 0 for two simulated datasets constructed as described above with *r* = 0.03 (Fig. 2a) and *r* = 0.1 (Fig. 2b). The variation in the *P_LRT_* with eight partitions is created solely by the selection of different partitions, because the datasets were the same for all analyses in the same panel. It is interesting that the variation in *P*-values is less for the more-highly autocorrelated dataset (*r* = 0.1, Fig. 2b), which is likely due to the greater correlations between *SSR_i_* from different partitions which makes the test scores from the separate partitions more similar. Finally, note that these results are for “small” datasets compared to those that the method was designed to analyze; the variation in *P*-values from different random partitions is less for larger remote-sensing datasets [9]. For smaller datasets, the analyses can be run multiple times with different random partitions, and the overall *P*-value is selected as the median of multiple values computed.

To compare the statistical results for different numbers (and hence sizes) of partitions, we use the same simulation model on a 60 x 60 pixel map to investigate the power to reject H_0_:*b* = 0 using a LRT when the true value is *b* = 10 Table 3). For 8 and 16 partitions, a subset of six partitions was chosen at random to compute the pairwise correlations between *SSR_i_* among partitions and *SSE_i_* among partitions (Eqs. 13,(14). The proportion of the simulations for which H_0_:*b* = 0 was rejected changed little with the number of partitions, *n*_p_ = 1, 2, 4, 8, and 16. This suggests that loss of power when partitioning data is insensitive to the number of partitions.

**Table 3:** Power of the partition method for a given number of partitions. Five-hundred simulations were performed on a 60 x 60 map using Eq. (21) with *b* = 10 and either moderate (*r* = 0.03) or strong (*r* = 0.10) spatial autocorrelation. The hypothesis H_0_:*b* = 0 was tested at a significance level of α = 0.05 for different numbers of partitions: 1, 2, 4, 8, and 16. Significance was determined using an F-test (for 1 partition, i.e., the entire dataset) and LRTs (for partitions *n_p_* = 2, 4, 8, and 16). The reported values are the proportions of the 500 simulations for which H_0_:*b* = 0 was rejected.

Number of partitions	*r* = 0.03	*r* = 0.10
1	0.722	0.188
2	0.732	0.196
4	0.724	0.192
8	0.716	0.196
16	0.718	0.200

### Spatiotemporal model

We performed a simulation study using the time-series model given in Eq. (18) on a map of 40 x 40 pixels. Each time series was 30 data points long, with moderate temporal autocorrelation (β*_l_* = 0.2). Spatial autocorrelation in the matrix **C** was assumed to have the form *v*(*d_lk_*) = (1 – *nugget*)exp(–(*d_lk_*/*r*)) for *l ≠ k*; thus, a proportion *nugget* of the error variance is “local” to a pixel. Spatial autocorrelation was assumed to be either moderate or strong (*r* = 0.03 or *r* = 0.1). We estimated *r* from the correlation among residuals, while *nugget* was estimated during the GLS spatial analysis via maximum likelihood. Thus, unlike the spatial example (Fig. 1), the spatiotemporal example included a parameter in matrix **C** that was estimated.

We assumed that the map was divided into 16 squares (10 x 10 pixels each) and assigned to four land-cover classes (e.g., Fig. 4 in [9]). The time trends in each of the four land-cover classes were given by *c*_1_ = 0, *c*_2_ = *c, c*_3_ = 2*c, c*_4_ = 3*c*, with values of *c* = 0, 0.04, ..., 0.20. We simulated 500 datasets for each parameter combination and tested the hypothesis that there were no differences in trends among land-cover classes, H_0_:*c*_1_=*c*_2_=*c*_3_=*c*_4_, and the hypothesis that there was no overall time trend, H_0_:*c*=0. We performed the test using GLS on the entire map. We also partitioned the map into eight random partitions and tested each separately. We then combined the tests either by selecting the partition with the lowest *P*-value and adjusting for multiple comparisons, or combining the tests as described in the section *Combining tests from the partitions*.

**Fig. 3:**
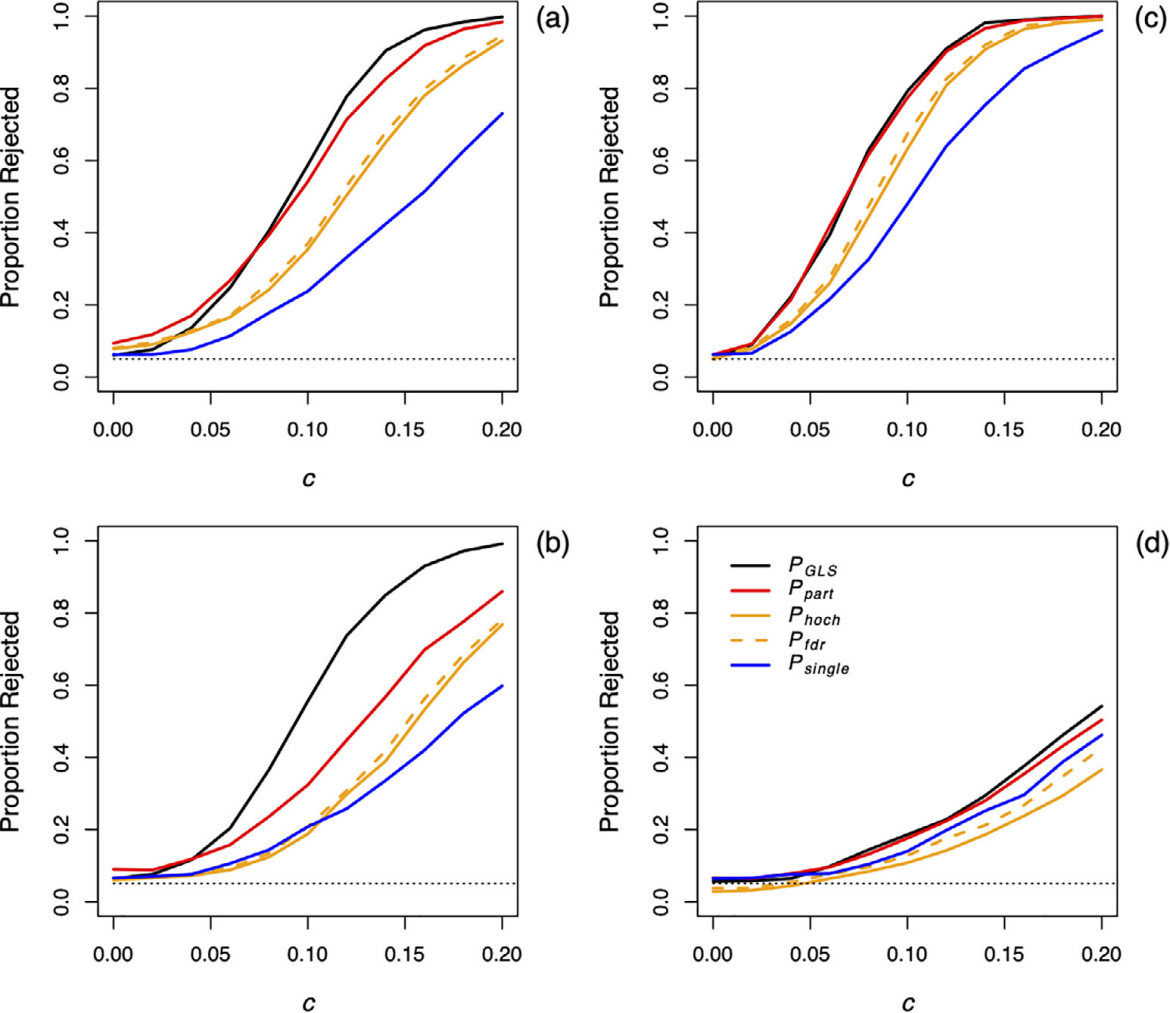
Type I errors and power for the methods combining correlated tests among partitions for spatiotemporal data. Five-hundred simulations were performed using Eq. (16), and the proportions of simulated datasets for which the null hypotheses of (a,b) no differences in time trends among land-cover classes and (c,d) no overall time trend were rejected at the significance level of α = 0.05. Simulations were performed for weak (*r* = 0.03; a,c) and strong (*r* = 0.1; b,d) spatial autocorrelation. The true time trends for the four land-cover classes were *c*_1_ = 0, *c*_2_ = *c, c*_3_ = 2*c, c*_4_ = 3*c*, with values of *c* = 0, 0.02, ..., 0.2. To scale the time trends, the time variable *t* ranged from 0 to 1 over the 30-year simulated time series, and the standard deviation σ of δ*_l_*(*t*) was 1. Therefore, a value of 0.12, for example, represents a change in the mean value of *z_l_*(*t*) over 30 years of 0.12σ(1 − β*_ι_*^2^)^–0.5^. To include pixel-scale (non-spatially autocorrelated) variation, we added non-spatial variation in the form of a Gaussian random variable with mean zero and variance 0.16 to *c* for each pixel. Simulations were performed for a 40 x 40 grid of pixels with weak temporal autocorrelation (β*_l_* = 0.2). The hypotheses were tested using (i) a GLS analysis applied to all of the data (*P_GLS_*), (ii) a GLS applied to eight partitions of the data with the log-likelihood ratios combined among partitions (*P_part_*), (iii) the lowest *P*-value from the GLS analyses of the eight partitions correcting for multiple comparisons using a Hochberg (*P_hoch_*) or False Discovery Rate (*P_fdr_*) adjustment, and (iv) a randomly selected partition (*P_single_*).

For both tests of differences among land-cover classes (Fig. 3a, b) and tests for an overall trend (Fig. 3c, d), the GLS analyses of the entire map had the highest statistical power to reject the null hypothesis. The method combining statistical results among partitions had the second-highest power (*P_part_*), while the method using adjustments for multiple comparisons had lower power (*P_hoch_* and *P_fdr_*). Finally, in general picking a single partition at random had the lowest power (*P_single_*). Spatial autocorrelation reduced the statistical power of all methods (*r* = 0.1; Fig. 3b, d). The method combining statistical results among partitions (*P_part_*) had somewhat inflated type I error rates for the analyses of land-cover classes, rejecting 9% of the simulated datasets when the null hypothesis was true. The inflated type I error rates, however, were the result of the relatively small size of the simulated map (40 x 40 pixels) necessitated by the application of the GLS analysis of the entire map (*P_GLS_*). Repeating the same analysis on a 60 x 60 pixel map (*P_part_*) gave rejection rates of 5.4% (*r* = 0.03) and 5.2% (*r* = 0.1). The inflated type I errors for the smaller map occurred due to the estimation of the *nugget*, because GLS in which no parameters in the correlation matrix **C** are estimated does not give inflated type I errors [10].

## Declaration of Competing Interest

The authors declare that they have no known competing financial interests or personal relationships that could have appeared to influence the work reported in this paper.
